# First experience with Tolvaptan for the treatment of neonates and infants with capillary leak syndrome after cardiac surgery

**DOI:** 10.1186/s12887-019-1418-6

**Published:** 2019-02-12

**Authors:** Anne Kerling, Okan Toka, André Rüffer, Hanna Müller, Sheeraz Habash, Christel Weiss, Sven Dittrich, Julia Moosmann

**Affiliations:** 1 Department of Pediatric Cardiology, University of Erlangen-Nürnberg, Loschgestrasse 15, 91054 Erlangen, Germany; 2 Department of Pediatric Cardiac Surgery, University of Erlangen-Nürnberg, Loschgestrasse 15, 91054 Erlangen, Germany; 3 Department of Pediatrics and Adolescent Medicine, University of Erlangen-Nürnberg, Loschgestrasse 15, 91054 Erlangen, Germany; 4 Department of Medical Statistics and Biomathematics, University Hospital Mannheim, University of Heidelberg, Theodor-Kutzer-Ufer 1-3, 68167 Mannheim, Germany

## Abstract

**Background:**

Postoperative fluid management in critically ill neonates and infants with capillary leak syndrome (CLS) and extensive volume overload after cardiac surgery on cardiopulmonary bypass is challenging. CLS is often resistant to conventional diuretic therapy, aggravating the course of weaning from invasive ventilation, increasing length of stay on ICU and morbidity and mortality.

**Methods:**

Tolvaptan (TLV, vasopressin type 2 receptor antagonist) was used as an additive diuretic in neonates and infants with CLS after cardiac surgery. Retrospective analysis of 25 patients with CLS including preoperative and postoperative parameters was performed. Multivariate regression analysis was performed to identify predictors for TLV response.

**Results:**

Multivariate analysis identified urinary output during 24 h after TLV administration and mean blood pressure (BP) on day 2 of TLV treatment as predictors for TLV response (AUC = 0.956). Responder showed greater weight reduction (*p* < 0.0001), earlier weaning from ventilator during TLV (*p* = 0.0421) and shorter time in the ICU after TLV treatment (*p* = 0.0155). Serum sodium and serum osmolality increased significantly over time in all patients treated with TLV.

**Conclusion:**

In neonates and infants with diuretic-refractory CLS after cardiac surgery, additional aquaretic therapy with TLV showed an increase in urinary output and reduction in bodyweight in patients classified as TLV responder. Increase in urinary output and mean BP on day 2 of treatment were strong predictors for TLV response.

**Electronic supplementary material:**

The online version of this article (10.1186/s12887-019-1418-6) contains supplementary material, which is available to authorized users.

## Introduction

Regulation of volume and electrolyte homeostasis after cardiac surgery on cardiopulmonary bypass (CPB) in newborns and infants with congenital heart defects (CHD) is challenging [1, 2]. The use of CPB during open heart surgery is accompanied by an inflammatory response leading to capillary leak syndrome (CLS) [3–5]. CLS can be defined by the clinical presentation of third space volume overload with consecutive generalized edema and substantial gain of weight, intravascular hypovolemia, hypoalbuminemia and hemoconcentration in the absence of severe congestive heart failure (CHF). In conjunction, an elevation of subcutaneous-thoracic ratio (ST-ratio) can help to diagnose CLS in the pediatric population [3, 4, 6, 7]. Prolonged interstitial fluid retention due to CLS is often resistant to conventional diuretic therapy, aggravating weaning from invasive ventilation, leading to longer time at the ICU and increasing postoperative morbidity (e.g. pulmonary infections) and mortality [4, 8, 9]. There have been great efforts in early detection and prevention of CLS [3, 10]. However, improvements in treatment strategies especially for neonates and children after cardiac surgery are still needed.

Tolvaptan (TLV) is a selective competitive vasopressin 2 receptor antagonist and prohibits the movement of aquaporin 2 into the luminal wall of the collecting duct and thereby reduces the reabsorption of water [11, 12]. TLV has been FDA (Food and Drug Administration) approved for the treatment of hyponatremia associated with CHF in adults and the syndrome of inappropriate antidiuretic hormone secretion (SIADH) in adults and children. The approval for treatment of hyponatremia in patients with liver cirrhosis was removed due to reported hepatotoxicity in adults, and the duration of treatment was limited to 30 days. TLV has also shown efficacy in treatment of autosomal-dominant polycystic kidney disease [12–16].

Several studies including a phase III study illustrated the efficacy of TLV in CHF with hypervolemia and hyponatremia especially during the acute phase of cardiac decompensation and diuretic resistance in adults [17–19]. The multicenter, retrospective J-SPECH study from 2015 suggested that TLV can be safely administered in pediatric patients but may be less effective in neonates and infants compared to adolescence or adults [14, 20]. Differences in the response profiles to TLV were often seen, however they had been unpredictable in the beginning. Recent studies defined TLV response as an increase of urine volume after its administration, patients responding with an increase are defined as responder [21, 22].

The role of TLV in postoperative fluid management after cardiac surgery on CPB has been evaluated in postoperative treatment in adults, but little is known about its role in infants and neonates [18, 23, 24]. One recent retrospective study in pediatric patients after uncomplicated cardiovascular surgery (shunt closure) compared treatment of additional TLV to patients treated with standard diuretic therapy [25]. TLV treatment was safely administered and resulted in an increase in urinary output, showing a potential reduction of intravenous loop-diuretic use during treatment course [25].

We used TLV in the postoperative fluid management in critically ill infants and neonates with postoperative CLS, massive volume overload and diuretic resistance after complex cardiac surgery and we retrospectively analyzed parameters to predict TLV response.

## Materials and methods

### Patients

Our retrospective analysis encompasses a single center experience (Department of Pediatric Cardiology at the Friedrich-Alexander-University of Erlangen-Nürnberg, Germany). We included 25 patients with CHD after cardiac surgery, treated with TLV in ICU between June 2011 and May 2017, evaluating effects of postoperative TLV therapy in patients with CLS. Criteria for the use of add-on therapy was 1) fluid overload 2) no increase in urinary output under conventional diuretic therapy 3) persisting renal function (no anuria) 4) low serum sodium. Descriptive patient’s auxologic and clinical characteristics, leading cardiologic diagnosis and the respective surgical procedures are displayed in Tables 1 and 2. Our cohort included four preterm patients (1: 31 + 6; 2: 35 + 6; 3: 34 + 5; 4: 34 + 5). Corrected age for preterm infants at TLV treatment was 35 + 3, 39 + 5, 41 + 0 and one infant received therapy 8 month after birth.

**Table 1:** Patient demographics

Parameter	CLS		*P*-value
	Responder	Non-responder	
n	17	8	
Female	9	4	1.0000
Male	8	4	
Gestagional age (SSW)	37 + 4 (31 + 6–40 + 1)	38 + 1(37 + 1–40 + 0)	0.1049
RACHS-1score	3 (2–6)	3 (2–6)	0.2253
Basic Aristotle score	10 (4–15)	7.25 (6.5–14.5)	0.0997
Cardiopulmonary bypass time (min)	219 (0–390)	148 (56–439)	0.1709
Cross clamp time (min)	77 (0–177)	51 (8–173)	0.3508
Subcutaneous-thoracic-ratio (%)	21.0 (14.3–26.5)	19.75 (14.7–27.9)	0.6408
Secondary chest closure after surgery (days)	8 (2–24) (*n* = 11)	10 (2–17) (*n* = 4)	0.9878
Start of TLV after surgery (days)	13 (2–44)	15 (7–24)	1.0000
Age when TLV therapy was started (days)	35 (9–228)	37.5 (20–549)	0.3821
Preoperative weight (kg)	3.30 (1.88–4.27)	3.20 (1.93–6.32)	0.7487
Absolute weight before TLV (kg)	4.35 (2.83–5.55)	4.42 (2.61–5.68)	0.8673
Weight above dry weight when TLV was started (%)	131.8 (102.6–202.8)	133.5 (113.5–154.4)	0.8151
Dose of TLV administration (mg/kg)	0.53 (0.15–1.06)	0.49 (0.13–0.95)	0.6204
Period of TLV administration (days)	8 (1–25)	7 (1–47)	0.6391
Urinary output 24 h prior to Tolvaptan administration (ml/kg/h)	4.15 (0.92–9.18)	3.27 (0.54–9.40)	0.4665
Urinary output 24 h after Tolvaptan administration (ml/kg/h)	6.38 (1.20–15.41)	2.21 (0.28–7.15)	0.0039
Days on ICU after TLV administration	15 (3–111)	40.5 (16–139)	0.0155
Death	1	3	0.0808

Frequencies are given for binary data; for quantitative and ordinal data median and range are presented. *p* < 0.05 has been considered as statistically significant

**Table 2:** Diagnosis and surgical procedures

Diagnosis	Operation	Responder	Non-responder	STS-EACTS mortality category	Major complications (95%CrI)
Aortic arch hypoplasia	Reconstruction of the aortic arch	1		3	12.2%
Dextro Transposition of the great arteries (d-TGA)	Arterial switch operation + ASD and/or VSD closure	4		3	10.7%
	Arterial switch operation + VSD patch and aortic arch repair	1		5	31.0%
Pulmonary atresia	RVOT patch- enlargement		1	2	6.5%
Single ventricle	Norwood	2		5	29.7%
	Bidirectional Glenn-anastomosis	1	1	2	6.4%
	aortic arch reconstruction	1	1	4	15.9%
	DKS anastomosis + aortic arch reconstruction	1		5	22.9%
Fallot-Tetralogy (TOF)	TOF repair (RVOT patch, VSD patch)	1	1	1	7.7%
	Blalock-Taussig-Shunt	1		4	12.4%
Interrupted aortic arch	aortic arch repair	1	1	4	19.0%
Double outlet right ventricle (DORV)	Closure of aorto-pulmonary-window	1		2	6.2%
	Norwood	1		5	29.7%
Tricuspid atresia Ib	Aorto-pulmonary shunt		1	4	11.3%
Total anomalous pulmonary venous return (TAPVC)	Correction of pulmonary vein anomalies	1	1	4	16.4%
Mitral valve insufficiency	Mitral valve reconstruction, Ring implantation		1	2	6.6%
Total		17	8		
Mean				3.4	15.3%
Responder				4	12.4%
Non responder				3	9.5%

Values are expressed as absolute frequencies for binary data

We evaluated Risk Adjusted Congenital Heart Surgery score (RACHS-1) [26, 27] and basic Aristotle score [28] to quantify risk and complexity of the performed surgeries. STS-EACTS mortality category and associated major complications (95% CrI) were implemented to express mortality associated with congenital heart surgery and classifying congenital heart surgery procedures on the basis of their potential for morbidity [29, 30]. Team of surgeons, anesthesiologists and pediatric cardiologists remained unchanged during the study period. All patients underwent median sternotomy. Post-operative treatment was exclusively supervised on the pediatric cardiology ICU, beat-to-beat circulatory and pulmonary status, fluid and electrolyte homeostasis was digitally monitored, clinical status and organ function was monitored and digitally documented routinely by critical care nursing staff. Co-medication including conventional diuretic therapy before and during the treatment course with TLV was analyzed.

### Definition of CLS, responder- and non-responder –grouping

CLS was defined by clinical symptoms (volume overload, intravascular hypovolemia, low total protein, hypoalbuminemia, hemoconcentration) and subcutaneous-thoracic ratio (ST-Ratio; > 97. percentile). ST-Ratio was evaluated to quantify CLS by chest x-ray with anterior-posterior beam path [6]. X-rays were documented with Web Ris (celsius37.com AG, Mannheim, Germany).

Responder to TLV were classified according to the definition from adult studies by Imamura et al. [21, 22, 31] “responders as patients with any increase in urine volume (UV) at day 1 when TLV administration was started”. To classify individuals as responder in our population to TLV we permitted an increase of > 10% in urinary output within 24 h after the first TLV administration. Others were classified as non-responder [20–22].

### Treatment protocol

TLV (“Samsca”, Otsuka, Japan) was administered as individual healing attempt in critically ill children with complicated postoperative course. Off-label use was explained and informed consent was obtained by all participating families. Starting dose of TLV was 25% of target dose (1 mg/kg/d). Dose finding was titrated based on clinical symptoms, side effects (see below) and serum sodium levels. Tablets are available in 15 mg and 30 mg. Provision of small dosages was performed by the department of pharmacology of the University hospital Erlangen. Tablets were pulverized and encapsulated. At the ICU the pulverized aliquots were diluted and administered via nasogastric tube.

### Definition of TLV related adverse events

Adverse events were retrospectively analyzed according to the criteria of Otsuka applying for the planned Phase 3b, multicenter study trial “effects of TLV in hospitalized children with euvolemic or hypervolemic serum hyponatremia”.

Adverse events are classified: 1) absolute serum sodium level > 145 mmol/L or an overly rapid rise in serum sodium level (an increase in serum sodium of > 8 mmol/L over a 10-h period, 12 mmol/L over a 24-h period. 2) neurological symptoms, or other signs or symptoms suggestive of osmotic demyelination. 3) worsening symptoms of hyponatremia. 4) elevations in AST or ALT that are > 2 x ULN (upper limit of normal) or levels that increase > 2 times their previously observed level.

### Data collection

TLV doses were calculated in mg/kg (preoperative weight)/d. Volume overload was quantified, assuming preoperative weight as 100%. TLV application period, time on mechanical ventilation, time until extubation, body weight, urinary output and total daily dose of selected concurrent medications were recorded by Integrated Care Manager (ICM, Drägerwerk AG & Co. KGaA, Lübeck, Germany) software solutions. Retrospective data acquisition of laboratory values before surgery, before TLV treatment and during TLV treatment was performed using Lauris (version 15.09.29.9, Swisslab GmbH, Berlin, Germany) (Table 1).

### Institutional protocol for transfusion and fluid management

Post-operative indication for transfusion was alike and followed our departmental transfusion algorithm: packed red blood cells (PRBC) were administered at a hemoglobin (Hb) level of 14 g/dl in cyanotic patients and 10 g/dl in non-cyanotic patients. In the case of on-going bleeding, fresh frozen plasma (FFP, 10-15 ml/kg) was transfused if quick reached below 50%. Platelets were transfused at a platelet count below 50 × 10^3^/μl.

Postoperative indication for fluid substitution of kristalloids (NaCl and Jonosteril) is central venous pressure (CVP) < 5, and low blood pressure (BP) according to age related reference ranges. Administration of colloidal volume expanders, i.e. albumin and hydroxyethyl starch (HAES) is performed in hemodynamically unstable cases or low serum albumin levels.

### Statistical analysis

Quantitative approximately normally distributed variables are expressed as mean ± standard deviation (SD). For ordinally scaled data (e.g. RACHS-1) and for variables with skewed distribution median value together with minimum and maximum are given. As most of variables in Table 1 (demographic parameters, co-medication and laboratory parameters) and 3 (co-medication and laboratory parameters) seem to be normally distributed and due to the rather small sample sizes non-parametric Mann-Whitney-U tests have been performed in order to compare the median values of the two groups. For qualitative factors (i.e. cardiac malformation or procedures) absolute frequencies are presented. Fisher’s exact tests have been used.

In order to investigate changes over time (regarding weight, serum sodium, osmolality, and urinary output) ANOVAs for repeated measurements have been performed including time point and responder group as fixed factors and patients’ ID as a random factor. For the liver enzymes, Friedman’s test was performed instead of ANOVA for repeated measurements, because of the skewed distribution. Multiple regression analysis including all parameters was performed to identify predictive parameters for TLV responder.

All statistical analyses were conducted using GraphPad Prism (version 6.05, GraphPad Software, Inc., La Jolla, CA 92037 USA) and SAS, release 9.4 (SAS institute Inc., Cary. NC, USA). The result of a statistical test has been considered as statistically significant if the *p* value was less than 0.05.

### Ethical statement

The retrospective study was approved by the ethics committee of the University of Erlangen-Nürnberg (Re.-No. 145_13B). The study was conducted in accordance with the Declaration of Helsinki [32].

## Results

### Demographics

Postoperative CLS was diagnosed in 25 patients after cardiac surgery. Clinical parameters to define CLS are displayed in Table 1.

According to the definition of TLV responder by Imamura et al. [21, 22, 31] 17 individuals were identified as responder to TLV defined by an increase in urinary output > 10% in 24 h and 8 infants were identified as non-responder [20–22] (Table 1).

Age was similar in both groups (median 35 and 37.5 days; *p* = 0.3821). The underlying cardiac malformation and surgical procedures are displayed in Table 2. Extracardiac malformations and syndromes were Trisomy 21 in one responder and one non-responder patient, Turner syndrome in one non-responder and omphalocele in one responder patient. Surgical parameters (cardio pulmonary bypass (CPB) time, cross clamp time and surgical risk scores RACHS-1 and Aristotele score) are displayed in Table 1. In 15 patients, primary chest closure was not possible and secondary closure was performed. Both groups presented with increased ST-ratio > 97. percentile (*p* = 0.6408). A significant positive correlation was identified between ST-ratio and time on CPB (*p* = 0.0305, Pearson‘s correlation coefficient *r* = 0.4333). STS-EACTS mortality category was 4 in responder and 3 in non-responder (*p* = 0.2201) and estimated major complication rates are 15.3% in responder compared to 12.4% in non-responder (*p* = 0.2190). Four responder patients showed severe infection with elevated procalcitonin (PCT) (*n* = 1 necroticing enterocolitis, *n* = 1 positive blood culture with Straphylococcus epidermidis, *n* = 1 pneumonia with *Enterococcus faecalis*, *n* = 1 *Enterococcus faecium* wound infection). Infection rates normalized before TLV treatment in all responder patients. One non-responder patient presented with an infection during treatment (*n* = 1 *Staphylococcus epidermidis* in intraoperative pericardial swab). Postoperative major complications are demonstrated in Table 3.

**Table 3:** Major complications

	Responder	Non-Responder	*p*-value
Postoperative acute renal failure requiring temporary dialysis			
Hemodialysis	1/17 (10 days)	1/8 (18 days)	1.0000
PD	6/17 (10 days; 3–19)	6/8 (10 days; 4–29)	0.1936/ 0.8099
Postoperative neurologic deficit	1/17	1/8	1.0000
Postoperative mechanical circulatory support	5/17 (8 days; 4–12)	5/8 (12 days; 7–34)	0.1936 /0.2073
Phrenic nerve injury	3/17	1/8	1.0000
Unplanned reoperation	2/17	3/8	0.2833

Major complications according to the Society of Thoracic Surgeons. Values are expressed as median and range. *p* < 0.05 has been considered as statistically significant.Fisher-Test was used to compare numbers of complications in both groups, Mann-Whitney-U-Test was used for differences between durations

Postoperative days on ICU, before TLV therapy was started (*p* = 1.0000), preoperative weight (*p* = 0.7487) and absolute weight (*p* = 0.8673) when TLV was started were not significantly different between responder and non-responder. All individuals presented with increased body-weight with a median of 131.8% over their preoperative weight in the responder group and 133.5% in the non-responder group, when TLV was started (*p* = 0.8151). The duration of TLV application (*p* = 0.6391) and average dose of TLV (*p* = 0.6204) administered were similar. Median length of stay in the ICU after TLV administration was significantly shorter in responder compared to non-responder patients (15 vs. 40.5 days; *p* = 0.0155).

We observed four deaths in the study population, one responder and three non-responder (*p* = 0.0808.) 17 days, 34 days, 35 days and 48 days after starting TLV.

Laboratory parameters were analyzed at several time points. Preoperative parameters did not show significant differences between both groups (Additional file 1: Table S1). Before TLV treatment non-responder group presented with a higher hematocrit (*p* = 0.0169) and higher hemoglobin level (*p* = 0.0168). According to CLS criteria: total protein was lowered in both groups (responder: 37.0 g/l and non-responder: 38.84 g/l; *p* = 0.9303) and median albumin levels were decreased in responder 20.15 g/l and non-responder 21.50 g/l (*p* = 0.7983). Serum sodium levels were low/normal in both groups (responder: 135 mmol/l vs. 130.5 mmol/l; *p* = 0.1269). No differences were observed for serum blood urea nitrogen (BUN), creatinine, potassium and serum osmolality before TLV treatment was started (Table 4).

**Table 4:** Co medication and laboratory parameters

	Responder	Non responder	*P*-value
Co-medication (mg/kg/day) when TLV was started			
Furosemide perfusor	5.93 (0.60–7.98) (*n* = 16)	5.57 (1.70–10.9) (*n* = 8)	0.1522
Thiazide oral	1.93 (0.99–3.72) (*n* = 12)	1.03 (0.46–2.09) (*n* = 5)	0.1703
Spironolactone oral	2.27 (0.93–6.38) (*n* = 13)	2.08 (2.05–2.78) (*n* = 4)	0.7339
Etacrynacid intravenous	1.09 (0.69–3.41) (*n* = 5)	1.15 (1.04–1.26) (*n* = 2)	1.0000
Laboratory parameters before TLV treatment			
Hematocrit (%)	40.07 (31.73–53.60)	44.08 (39.87–51.37)	0.0169
Hemoglobin (g/dl)	13.12 (9.5–19.02) (*n* = 16)	14.25 (13.40–16.83)	0.0168
Total protein (g/l)	37.0 (27–45.67)	38.84 (31.67–44.00)	0.9303
Albumin (g/l)	20.15 (18.43–26.40) (*n* = 7)	21.50 (17.8–25.0) (*n* = 7)	0.7983
serum BUN (mg/dl)	42.00 (7.67–113.50)	50.83 (24.33–109.17)	0.4316
Creatinine (mg/dl)	0.44 (0.24–1.47)	0.66 (0.25–1.02)	0.4484
Sodium (mmol/l)	135 (129–144)	130.5 (126.17–137.67) (*n* = 7)	0.1269
Potassium (mmol/l)	4.10 (3.63–4.80)	4.09 (3.80–4.60)	0.8156
Osmolality (mosm/kg)	281.67 (267.5–307.0) (*n* = 16)	280.92 (261.0–291.0)	0.4258

For quantitative and ordinal data median and range are presented. *p* < 0.05 has been considered as statistically significant

Vital parameters including (BP, heart rate (HR) and CVP) were analyzed. CVP decreased during TLV treatment in both groups, but was not significantly different. Mean BP was lower in non-responder on day 2 (*p* = 0.0035) and day 3 (*p* = 0.0309) of treatment (Additional file 1: Table S2).

#### Predicting TLV response

Multivariate regression analysis to predict TLV response revealed mean BP on day 2 of TLV administration and urinary output 24 h after TLV as significant combined predictors for responder to TLV. Predicting TLV response with an AUC = 0.956.

The probability for TLV response increases by 1.185 / mmHg mean BP on day 2 of TLV treatment and the probability for TLV response increases by factor 2.064 / ml/kg/h urinary output after 24 h after TLV administration.

Mathematical model to estimate the probability for responder:

$$ \mathrm{probability}\ \mathrm{for}\ \mathrm{response}\ \mathrm{to}\ \mathrm{TLV}=\frac{\mathit{\exp}\left(-12.34+0.1696\times mean\  bp\  day\ 2\  of\  TLV+0.7248\times {}^{"} urinary\ output\ 24h\  after\  TLV\right)}{1-\left(\mathit{\exp}\left(-12.34+0.1696\times mean\  bp\  day\ 2\  of\  TLV+0.7248\times {}^{"} urinary\ output\ 24h\  after\  TLV\right)\right)} $$

#### Tolvaptan effects on bodyweight, serum sodium levels, osmolality and urinary output

**Fig. 1:**
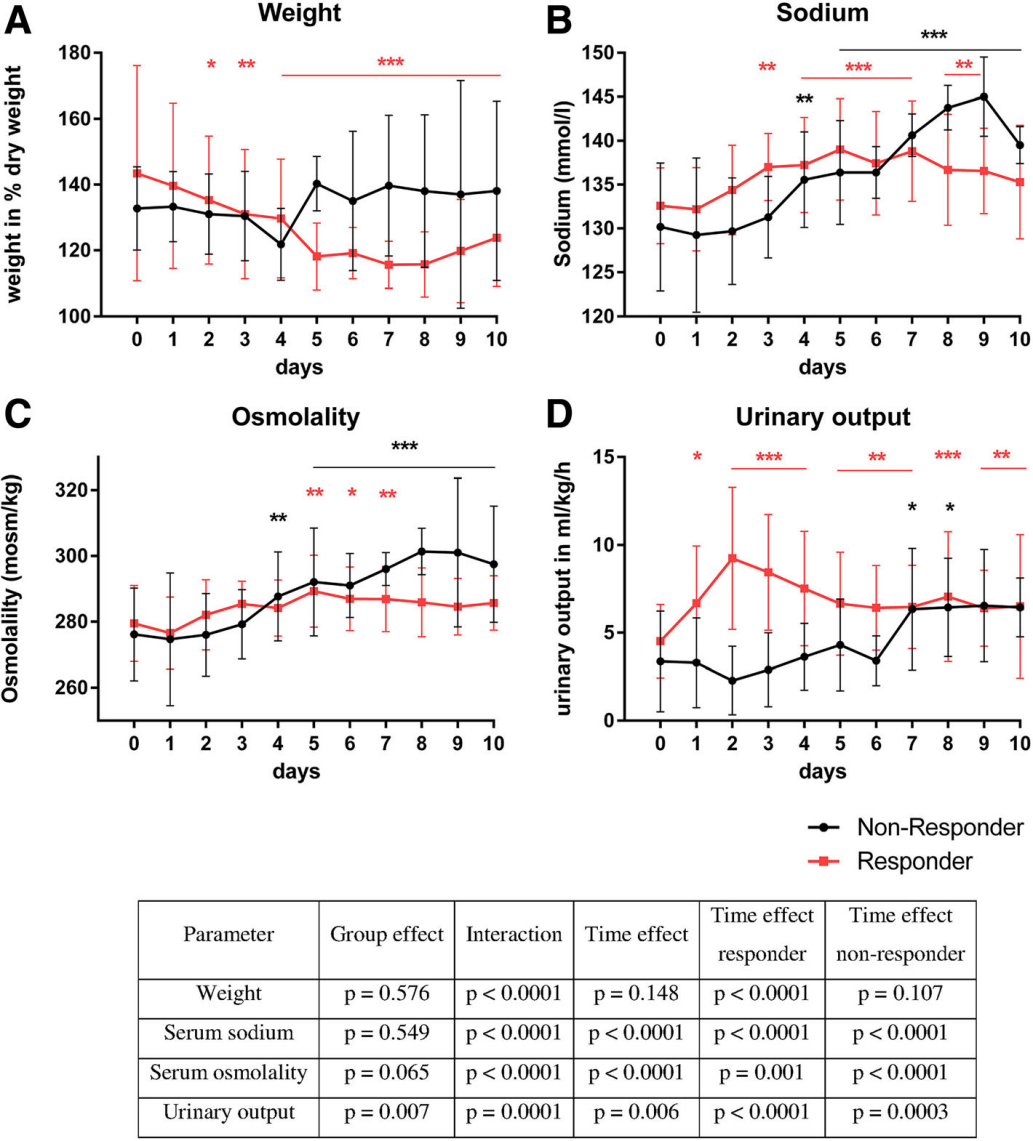
Weight (**a**), serum sodium (**b**), serum osmolality (**c**) and urinary output (**d**) during 10 days of TLV treatment. Responders (red graph) and non-responder (black graph); * *p* < 0.05 (related to day 0) ** *p* < 0.01 (related to day 0) *** *p* < 0.001 (related to day 0). *p*-values deriving from 2 way ANOVAs; *p* values for time effect deriving from 2 separate ANOVAS for responders and non-responders. Changes over time regarding bodyweight, serum sodium, osmolality and urinary output have been tested using ANOVAs for repeated measurements with group (responder / non-responder) and time point as fixed factors. The p-values in Table 2 reveal that for each parameter interactions between group and time effects could be observed indicating that response profiles of the two groups differ (see Fig. 1a and d)

For each parameter (bodyweight, serum sodium, osmolality and urinary output) and for each group (responders, non-responders) changes over time could be observed (with the only exception for the weight parameter in the non-responder group) (Fig. 1a-d).

Responders showed a significant weight reduction starting at day # 2 after TLV administration. The greatest weight reduction was achieved at day # 7 of treatment down to 115.6 ± 7.1% (*p* < 0.0001) of preoperative weight. Fig. 1a shows the weight progression between responder and non-responder group over 10 days of TLV administration. Non-responder did not show a significant weight reduction in the investigated time period (*p* = 0.1067), while responders showed a significant weight reduction (*p* < 0.0001) (Fig. 1).

Urinary output 24 h after the first dose of TLV was significantly higher (by definition of responder) in the responder group (*p* = 0.0039; Table 1; Fig. 1d). During all 10 days of treatment urinary output stayed higher (related to day 0) in the responder group. In the non-responder group urinary output also increased over the total investigated time period (*p* = 0.0003), but a significant increase from day # 0 was later than in the responder group on day 7 and 8 of treatment (Fig. 1d).

Before TLV therapy, responder and non-responder presented with median serum sodium at the lower cut off to normal. A significant increase was identified during the investigated time period in both groups (*p* < 0.0001) (Fig. 1b). No significant difference between responder and non-responder groups was observed (*p* = 0.5489, accumulated over time), however the response profiles were different (*p* < 0.0001). In responder, a significant increase of serum sodium was seen at day # 3, in non-responder at day # 4. In the responder group, hypernatremia was not observed. We observed one adverse event related to TLV in the non-responder group, one patient developed hypernatremia (151 mmol/l) on day # 9, which was reversible on the following day.

Osmolality increased in both groups over treatment course (non-responder *p* < 0.0001 and responder *p* = 0.001) (Fig. 1c). Significant changes in osmolality were seen on day # 4 in the non-responder and on day # 5 in the responder group (Fig. 1c).

#### Liver metabolism

Liver enzymes were monitored before, during and after TLV treatment course. Due to the limitations of retrospective data analysis measurements were not performed on a regular basis of a distinct study protocol. Regarding the upper cut off values of alanine- aminotransferase (ALT; normal < 26 U/l), aspartate- aminotransferase (AST; normal < 50 U/l) and Gamma-Glutamyltransferase (GGT; normal < 23 U/l), 3/8 of the responder, 4/8 of the non-responder presented with significantly elevated GGT before TLV treatment, already. 2/8 of non-responder presented with initial AST elevation. ALT elevation was present in 3/8 of the non-responder. In both groups no significant elevation of AST, ALT and GGT was identified for median group parameters during and after treatment (Table 5).

**Table 5:** Liver metabolism

Group	Parameter	Before TLV	Under TLV	After TLV	*P*-value
Responder (*n* = 17)	GGT (< 23 U/l)	41.5 (19–93)*n = 8*	95.5 (29–215) *n = 6*	118 (38–354) *n = 9*	0.2926
Non-responder (*n* = 8)		53.5 (17–220)	93 (34–352) *n = 7*	116.5 (24–845) *n = 6*	0.2223
Responder (*n* = 17)	AST (< 50 U/l)	22.5 (10–48) *n = 8*	20.5 (10–57) *n = 8*	23.5 (14–143) *n = 10*	0.4576
Non-responder (*n* = 8)		32.5 (11–638)	57 (14–252) *n = 6*	28.5 (13–50)	0.1677
Responder (*n* = 17)	ALT (< 26 U/l)	16 (9–30) *n = 7*	14 (6–178) *n = 9*	17.5 (10–26) *n = 8*	0.4987
Non-responder (*n* = 8)		30.5 (11–382)	51 (7–399) *n = 7*	21.5 (6–107)	0.1303

Normal values for GGT, AST and ALT are expressed. Values are expressed as median and range. *p* < 0.05 has been considered as statistically significant

#### Co-medication, transfusions and fluid management

Diuretic and catecholamine therapy before surgery is listed in Additional file 1: Table S1 presenting no differences between both groups. Postoperative catecholamine therapy and diuretic treatment before TLV administration was not different between responder and non-responder (Additional file 1: Table S3). Intravenous additional diuretic therapy could be reduced in both groups during treatment course with TLV (by factor 3.68 and 3.77, respectively). An ANOVA for repeated measurements revealed no statistical difference between the responders and non-responders (*p* = 0.3935) and no statistically significant interaction term (*p* = 0.6127). However, reduction over the investigated time could be observed in both groups (*p* < 0.0001). Nephrotoxic medication (i.e. vancomycin, fluconazole and tobramycin) was administered in a subset of patients in both groups, no differences were observed (Additional file 1: Table S3). Estimated glomerular filtration rate (GFR; by Schwartz formula) before and during treatment is provided in Table 6. All patients received postoperative kristalloids, substitution during TLV and after TLV is listed in Additional file 1: Table S3 and did not show differences between both groups. Only a very limited number of patients received kolloids, mainly albumin. HAES was only substituted in two non-responder patient during the immediate postoperative course (Additional file 1: Table S3).

**Table 6:** Airway management and GFR

	Responder	Non-responder	*p*-value
Mechanical ventilation	17	7	0.3200
Non-invasive ventilation	0	1	0.3200
Extubation during TLV	10	1	0.0421
Extubation 2–7 days after TLV	2	0	1.0000
Extubation > 7 days after TLV	3	3	0.3442
No Extubation and tracheostoma	2	3	0.2833
Oxygenation index			
Postoperative	173 (45–365)	137 (38–253)	0.0857
Before TLV	235 (127–366)	139 (83–284)	0.0702
1st day	241 (103–377)	162 (132–243)	0.1829
2nd day	264 (110–355)	181 (103–272)	0.0524
3rd day	209 (98–374)	139 (125–266) *n* = 5	0.4274
Before extubation	255 (167–355)	214 (118–331) *n* = 3	0.5708
Glomerular filtration rate (GFR)			
Preoperative	37 (28–101)	39 (15–114)	0.8531
Postoperative	34 (25–81)	36 (22–73)	0.4833
Before TLV	41 (17–97)	38 (20–108)	0.5774
5 days after begin of TLV	49 (22–105)	39 (20–62)	0.2811

Frequencies are given for binary data; for quantitative and ordinal data median and range are presented. *p* < 0.05 has been considered as statistically significant

#### Airway management

Mechanical or non-invasive ventilation was required in all CLS patients (Table 6). All 17 responder patients needed mechanical ventilation before TLV administration. In 10 patients, invasive ventilation could be ended during TLV administration. In 2 responder patients, extubation was performed within 7 days after TLV. 3 individuals were extubated more than 7 days after TLV treatment course. 2 patients were not extubated and received a tracheostoma. In the non-responder group, 7 patients required mechanical ventilation, only one of them could be extubated during treatment course with TLV. Horovitz-index (oxygenation index) demonstrates improvements of respiration therapy and increased during treatment (Table 6). It was higher in responder compared to non-responder but did not show significant differences. Rate of extubation during TLV treatment was higher in responder compared to non-responder (*p* = 0.0421, Table 6).

## Discussion

We report a retrospective analysis of our single center experience with TLV treatment in infants and neonates after cardiac surgery with postoperative CLS to predict TLV response. Additional diuretic therapy with TLV increased urinary output > 10% in 2/3 of patients with CLS. According to the definition of Imamura et al. [21, 22, 31] patients with increased urinary output during the first 24 h, were classified as responder to TLV and presented with significant reduction in body weight. Increase in urinary output during the first 24 h after TLV administration and higher mean BP on day 2 of TLV treatment were identified as predictive factors for TLV response (AUC = 0.956).

The underlying mechanisms of TLV response have been studied in detail; TLV is able to antagonize antidiuretic hormone (ADH) overstimulation and thus increases urinary output due to aquaresis. ADH excretion can be triggered by intravascular hypovolemia, activation of renin angiotensin aldosterone (RAAS) axis (mainly angiotensin II, by chronic extensive diuretic abuse), reduced osmotic pressure (plasma osmolality <275mmosm/kg), stress and post-operative pain [33, 34]. All these parameter can be observed in pediatric patients with CLS due to long CPB time, presenting with third space volume overload and intravascular volume depletion and therefore no severe hyponatremia but low to normal serum sodium levels. In contrast, patients with postoperative renal or cardiac failure presenting with volume overload and intravasal hypervolemia and low serum sodium. We observed that the aquaretic TLV is not only effective in patients with hyponatremia and volume overload due to e.g. cardiac failure as shown earlier, but in especially in small neonates and infants with CLS including massive volume overload in the third space and almost normal sodium levels. In this group the additional aquaresis mobilized the volume from the third space and increase urinary output. In patients with intravasal hypervolemia and low serum sodium, intravascular volume is mobilized. In patients with CLS serum osmolality remains steady, supporting this physiologic hypothesis.

Diverse parameters are discussed to predict the response profiles to TLV however a gold standard has not been established [22, 35, 36]. Especially for our study population of neonates and infants no detailed criteria or predictors for TLV response are known. Thus, one aim of this study was to identify predictors for TLV response in this patient population. In our study cohort we identified urinary output during the first 24 h and mean BP on day 2 of TLV treatment as good predictors for TLV response. Patients presenting with an increase of urinary output by 1 ml/kg/h, the probability for TLV response increases by factor 2.1. Further, higher mean BP on day 2 increases the probability of factor 1.2 by each mmHg. Taken together, both parameters represent strong predictors for TLV response.

One potential explanation could be that increased mean BP at the beginning of TLV therapy in combination with the mechanisms of TLV described above supported and increased TLV effect leading to increased urinary output. On the other side, all other parameters including electrolytes and renal parameters (creatinine, BUN), fluid substitution, transfusions and concomitant medication etc. are not regarded as predictors after multiple regression analysis.

Nevertheless, statements about renal function and GFR are of limited power while using Schwartz formula which is critically discussed as valid parameter for calculating neonatal GFR. Cystatin C which was not routinely measured seems a more predictable parameter to estimate GFR in this patient population. The influence of other potential confounders such as (e.g. PD, adjunctive medication) cannot be completely ruled out, partly due to limited number of patients.

Most likely the response to TLV is also influenced by age, concomitant medication and degree of heart failure. As our study has some limitations because of its retrospective study design and because of the low sample size further studies to identify LTV predictors are necessary.

When comparing responder and non-responder: responder patients presented with significant reduction in body weight and reduction of additional standard diuretic during the TLV treatment course. Further, responder patients showed an improvement of their clinical course by earlier weaning from the ventilator and shorter time on ICU. Nevertheless, these parameters need critical evaluation in a randomized and blinded trial including an untreated control group to validate a positive effect of TLV on outcome parameters.

In the responder group the main effect of TLV treatment was noticeable during the first 5–6 days. Short-term treatment after cardiovascular surgery might be advantageous compared to long-term treatment due to a discussed TLV escape [13]. In patients who do not show an increase in urinary output (non-responder) a longer treatment should be critically discussed and possibly terminated to reduce potential side effects of TLV in pediatric population.

Side effects of TLV are well described by Otsuka Pharmaceutical and in the literature for adult patients. Nevertheless, pharmacodynamics in children and infants can differ severely from adults and randomized trials are missed in the pediatric population. Despite safety of TLV therapy was not the aim of the study: in our evaluation described side effects were retrospectively analyzed between the two subgroups. TLV was well tolerated particularly in terms of excessive sodium elevations or severe deterioration of liver function which did not occur. We had one case of hypernatremia which was reversible after one day. All patients receiving TLV showed high morbidity and mortality, therefore adverse effects especially on renal and cardiac impairment and long-term outcome could not be evaluated and need further evaluation in a prospective, randomized and blinded trial including an appropriate control group to validate a positive effect of TLV on outcome compared to standard care.

## Conclusion

The use of TLV added to conventional diuretic therapy in infants and neonates after cardiac surgery with CLS was effective in 68% of our patients with CHD and CLS after cardiac surgery. Responder to TLV presented with increase in urinary output and significant weight reduction. Reduction of diuretic co-medication was possible. Increase in urinary output during 24 h after TLV treatment and mean BP on day 2 of treatment were strong predictors for TLV response. Prospective, controlled and multicenter studies are desirable and needed to confirm the beneficial effects of TLV and to monitor side effects in the field of pediatric cardiology and neonates.

## Additional file

Additional file 1: **Table S1.** Preoperative data. **Table S2.** Vital parameters. **Table S3.** Catecholamine therapy, fluid management and transfusion management after surgery. (DOCX 20 kb)

## Abbreviations

ADH: Antidiuretic hormone
ALT: Alanine Aminotransferease
AST: Aspartate Aminotransferase
AUC: Area under the curve
BP: Blood pressure
BUN: Blood urea nitrogen
CHF: Congestive heart failure
CLS: Capillary leak syndrome
CPB: Cardio pulmonary bypass
CVP: Central venous pressure
FDA: Food and Drug Administration
FFP: Fresh frozen plasma
GFR: Glomerular filtration rate
GGT: Gamma-Glutamyltransferase
HAES: Hydroxyethyl starch
Hb: Haemoglobin
Hk: Hematocrit
HR: Heart rate
ICU: Intensive care unit
PCT: Procalcitonin
PRBC: Packed red blood cells
RAAS: Renin-angiotensin-aldosterone-system
SD: Standard deviation
SIADH: Syndrome of Inappropriate Antidiuretic Hormone Secretion
ST-ratio: Subcutaneous-thoracic ratio
STS-EACTS: Society of Thoracic Surgeons-European Association for Cardio- Thoracic Surgery
TLV: Tolvaptan
ULN: Upper limit of normal
UV: Urine volume
