# Supplementary material for: First experience with Tolvaptan for the treatment of neonates and infants with capillary leak syndrome after cardiac surgery
Source: BMC Pediatr. 2019 Feb 12;19:57. doi: 10.1186/s12887-019-1418-6 (PMC6371520; doi:10.1186/s12887-019-1418-6)
Supplement: Supplementary file 1 — Table S1. Preoperative data. Table S2. Vital parameters. Table S3. Catecholamine therapy, fluid management and transfusion management after surgery. (DOCX 20 kb) [file 12887_2019_1418_MOESM1_ESM.docx]

Additional file 1: **Table S1** Preoperative data.

| **Parameter** | **Responder** | **Non-responder** | **p-value** |
| --- | --- | --- | --- |
| Hematrocrit | 43.8  (33.2 – 51.1) | 43  (29.4 – 55.4) | 0.7932 |
| Hemoglobin | 14.1  (10.2 - 17) | 14  (9.8 – 18.2) | 0.6618 |
| BUN | 16  (7 – 42) | 28  (5 – 48) | 0.1895 |
| Creatinine | 0.55  (0.23 – 0.95) | 1  (0.25 – 1.23) | 0.7706 |
| Serum sodium | 135  (126 – 142) | 135  (126 – 141) | 0.5386 |
| **Diuretic treatment** | | | |
| Furosemid i.v. | 1/17 | 1/8 | 1.000 |
| Furosemid p.o. | 10/17 | 4/8 | 1.000 |
| Spironolactone p.o. | 1/17 | 1/8 | 1.000 |
| Thiazide p.o. | 1/17 | 1/8 | 1.000 |
| **Catecholamine treatment** | | | |
| Dopamine | None | 1/8  (6 days) | 0.3200 |
| Epinephrine | None | 2/8  (3 and 11 days) | 0.0933 |
| Milrinone | None | 2/8  (3 and 11 days) | 0.0933 |

Frequencies are given for binary data; for quantitative and ordinal data median and range are presented. Numbers expressing patients receiving treatment and median number of interventions including range. Significances were calculated using Fisher-Test and Mann-Whitney-U-Test.

**Table S2.** Vital parameters.

|  | **Responder** | **Non-Responder** | **p-value** |
| --- | --- | --- | --- |
| **Before surgery** | | | |
| **Blood pressure**  **Sys**  **Dia**  **Mean** | 72 (52-90)  42 (25-46)  53.5 (37-64) | 79 (66-89)  40 (37-53)  58 (42-69) | 0.3691  0.5599  0.5616 |
| **Heart rate** | 149 (128-179) | 144 (131-161) | 0.5263 |
| **After surgery** | | | |
| **Blood pressure**  **Sys**  **Dia**  **Mean** | 65 (51-91)  44 (28-60)  51 (41-63) | 67 (51-74)  48 (36-58)  55 (42-63) | 0.5240  0.3406  0.5014 |
| **Heart rate** | 156 (127-171) | 144 (126-169) | 0.1533 |
| **ZVD** | 14 (9-31) | 14 (9-26) | 0.6705 |
| **Before TLV (one day before)** | | | |
| **Blood pressure**  **Sys**  **Dia**  **Mean** | 78 (60-104)  45 (32-58)  58 (45-75) | 73 (58-96)  42 (27-64)  51 (44-70) | 0.3659  0.5991  0.3660 |
| **Heart rate** | 147 (116-179) | 153 (124-165) | 0.9072 |
| **ZVD** | 18 (11-39) | 17 (13-20) | 0.9103 |
| **1^st^ day of TLV** | | | |
| **Blood pressure**  **Sys**  **Dia**  **Mean** | 81 (61-116)  45 (35-63)  57 (49-74) | 81 (60-98)  42 (31-53)  53 (47-67) | 0.3505  0.2094  0.1287 |
| **Heart rate** | 146 (112-185) | 146 (128-163) | 0.4314 |
| **ZVD** | 17 (12-24) | 18 (14-22) | 0.6799 |
| **2^nd^ day of TLV** | | | |
| **Blood pressure**  **Sys**  **Dia**  **Mean** | 83 (64-107)  46 (31-62)  62 (42-76) | 71 (50-93)  39 (33-51)  50 (42-65) | **0.0411**  0.1015  **0.0035** |
| **Heart rate** | 151 (120-192) | 147 (120-164) | 0.9303 |
| **ZVD** | 15 (11-21) | 18 (14-21) | 0.2995 |
| **3^rd^ day of TLV** | | | |
| **Blood pressure**  **Sys**  **Dia**  **Mean** | 86 (62-103)  43 (31-62)  62 (44-71) | 75 (62-102)  41 (32-60)  49 (45-71) | 0.2933  0.1219  **0.0309** |
| **Heart rate** | 148 (122-185) | 148 (126-164) | 0.8841 |
| **ZVD** | 15 (10-22) | 16 (12-22) | 0.3393 |

For quantitative and ordinal data median and range are presented. p<0.05 has been considered as statistically significant.**Table S3.** Catecholamine therapy, fluid management and transfusion management after surgery.

|  | **responder** | **non-responder** | **p-value** |
| --- | --- | --- | --- |
| **Fluid management** | | | |
| **Kolloids (albumin, HAES)**  Postoperative  During TLV | 5/17  2/17 | 4/8  2/8 | 0.3942  0.5700 |
| **Kristalloids (NaCl, Jonosteril)**  Postoperative  During TLV  After TLV | 17/17  6/17  8/17 | 8/8  6/8  7/8 | n.a.  0.0968  0.0875 |
| **Postoperative catecholamine therapy** | | | |
| Dopamine  Dobutamine  Epinephrine  Norepinephrine  Mirlinone | 5/17  2/17  17/17  13/17  17/17 | 2/8  0/8  8/8  7/8  8/8 | 1.0000  0.5467  n.a.  0.6279  n.a. |
| **Postoperative blood management** | | | |
| Postoperative transfusion  PRBC  PC  FFP | 17/17 (5: 2-23)  11/17 (4: 1-29)  15/17 (5: 1-84) | 8/8 (10: 4-16)  8/8 (5: 1-19)  8/8 (15.5: 1-29) | n.a. / 0.4800  0.1292 / 1.0000  1.0000 / 0.2566 |
| **Nephrotoxic medication** | | | |
| Vancomycin  Tobramycin  Fluconazole | 9/17  3/17  1/17 | 4/8  2/8  1/8 | 1.0000  1.0000  1.0000 |

Numbers expressing number of patients receiving treatment and median number of interventions including range. Significances were calculated using Fisher-Test and Mann-Whitney-U-Test.PC= platelet concentrate; PRBC= packed red blood cells ; FFP = fresh frozen plasma; HAES = hydroxyethyl starch
